# Homer2 and Alcohol: A Mutual Interaction

**DOI:** 10.3389/fpsyt.2017.00268

**Published:** 2017-11-30

**Authors:** Valentina Castelli, Anna Brancato, Angela Cavallaro, Gianluca Lavanco, Carla Cannizzaro

**Affiliations:** ^1^Department of Sciences for Health Promotion and Mother and Child Care “G. D’Alessandro”, University of Palermo, Palermo, Italy

**Keywords:** alcohol, Homer proteins, Homer2, glutamate, addiction

## Abstract

The past two decades of data derived from addicted individuals and preclinical animal models of addiction implicate a role for the excitatory glutamatergic transmission within the mesolimbic structures in alcoholism. The cellular localization of the glutamatergic receptor subtypes, as well as their signaling efficiency and function, are highly dependent upon discrete functional constituents of the postsynaptic density, including the Homer family of scaffolding proteins. The consequences of repeated alcohol administration on the expression of the Homer family proteins demonstrate a crucial and active role, particularly for the expression of Homer2 isoform, in regulating alcohol-induced behavioral and cellular neuroplasticity. The interaction between Homer2 and alcohol can be defined as a mutual relation: alcohol consumption enhances the expression of Homer2 protein isoform within the nucleus accumbens and the extended amygdala, cerebral areas where, in turn, Homer2 is able to mediate the development of the “pro-alcoholic” behavioral phenotype, as a consequence of the morpho-functional synaptic adaptations. Such findings are relevant for the detection of the strategic molecular components that prompt alcohol-induced functional and behavioral disarrangement as targets for future innovative treatment options.

## Introduction

Chronic and excessive alcohol consumption is associated with several neuropsychiatric effects, such as impairment in judgment, learning, memory, perception, and motor coordination (1, 2) characterized by aberrant morpho-functional plasticity at the synaptic level (3). However, the neurobiological mechanism underpinning the pharmacological properties of alcohol and its biologically active adduct is complex and not completely elucidated yet, thus hampering the development of successful pharmacological therapies (4–10). In the central nervous system, a large variety of signals and neurotransmitters [e.g., dopamine (DA), serotonin, glutamate, GABA, opioids, endocannabinoids] play a prominent role in mediating the behavioral and pharmacological effects of alcohol (11–21). Most of them converge on the mesolimbic DA reward circuitry (22–25), a main hub in the mediation of the reinforcing effects of drugs of abuse. At a molecular level, alcohol, as well as its first metabolite acetaldehyde, has been shown to directly affect DA neurotransmission in the mesolimbic system by increasing neuronal firing in the ventral tegmental area and stimulating DA release in the nucleus accumbens shell (NACsh) (26–28). Beside DA transmission, the endogenous opioid and the endocannabinoid systems have been reported to contribute to alcohol-related behaviors, in particular, voluntary consumption, reward and relapse behavior, both in preclinical models (29) and in humans (30, 31). Both modulatory pathways fine tune incoming inputs to the limbic brain regions (32, 33) and, among them, primarily the excitatory amino acid glutamate efferents (34, 35).

Intact glutamate signaling is critical for motivation and reward, learning and memory, and cognitive performance, just those functions that are essentially altered in alcohol-dependent individuals (21, 36). Alcohol exposure, in fact, reshapes both pre- and postsynaptic excitatory neurotransmission within mesolimbic structures, prompting a hyperglutamatergic state that contributes to the development and expression of alcohol dependence (37–39). The discrete functional components of the postsynaptic density (PSD) at the glutamatergic synapse, in particular, contribute to the cellular localization, trafficking, and efficiency of the glutamatergic receptor subtypes. Alterations in their expression within the NAC and amygdala (Amy) might gate the development of alcohol-induced behavioral plasticity, focusing on PSD components as crucial interceders of the acute and chronic effects of alcohol (34, 40). Notably, among the postsynaptic scaffolding proteins, the Homer family has been largely characterized and highlighted as a primarily plasticity-conditioning element (41–46). In particular, preclinical and clinical data point to Homer2 isoform proteins as essential and active players in the expression of alcohol-induced behavioral and cellular plasticity (44, 47–51).

This review summarizes the physiological role played by Homer proteins as a whole, with an emphasis on the most recent evidence on Homer2 isoforms. In particular, we will show the key role played by Homer2 isoforms within the glutamatergic signaling in the modulation of the acute behavioral and neurochemical sensitivity to alcohol, the development of alcohol-induced neuroplasticity, as well as other pathological phenotypes associated with alcohol addiction.

## The Homers

**Figure 1:**
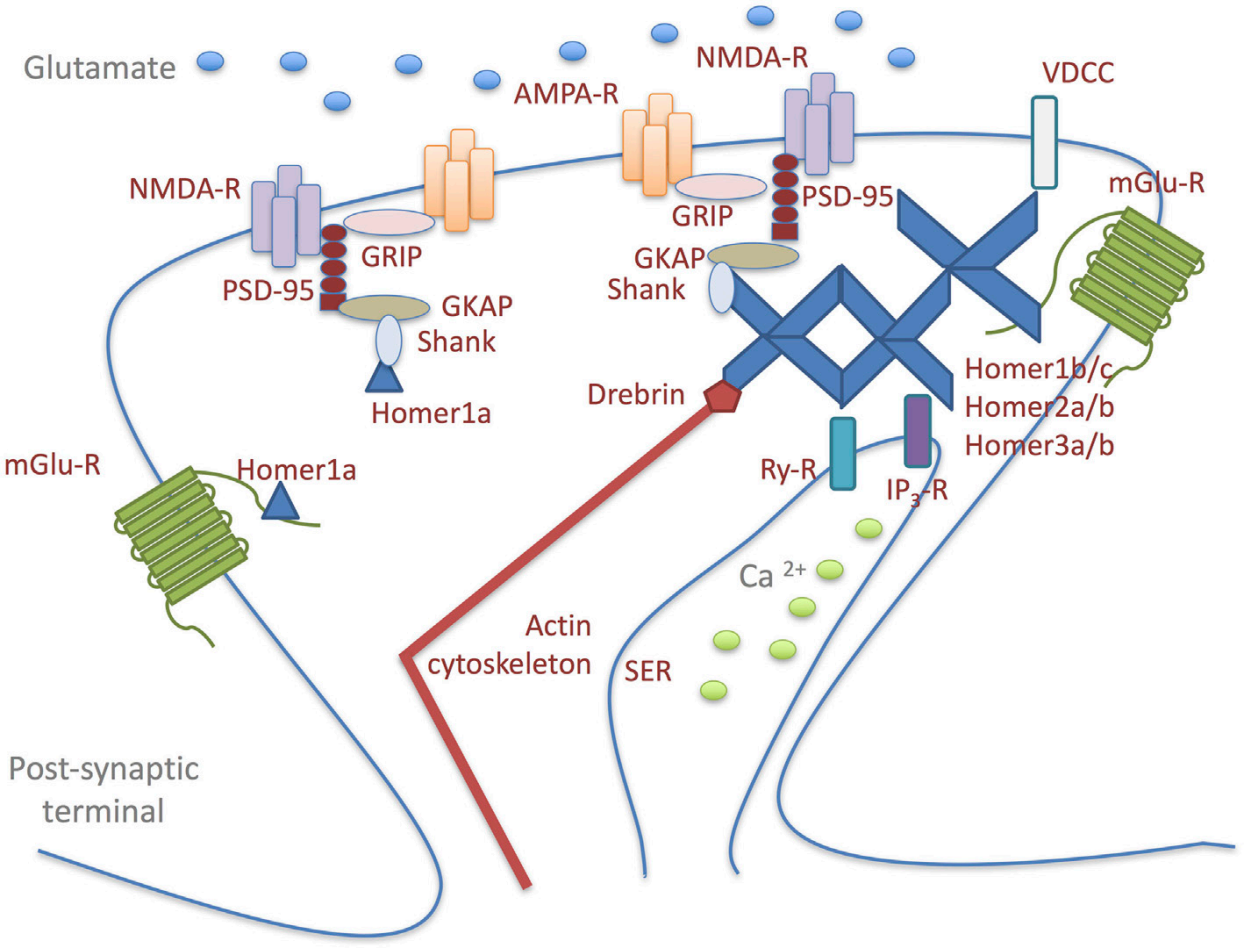
Homer proteins form a physical link among signaling molecules in glutamatergic postsynaptic density (PSD). Long Homer forms (Homer1b/c; Homer2a/b; Homer3a/b) bind to each other through their carboxy-terminal domains and to the target proteins: Group1 of metabotropic glutamate receptors (mGluRs); Ca^2+^-signaling-related proteins, including phospholipase C-ß (PLCß), inositol 1,4,5-trisphosphate receptors (IP_3_-R), ryanodine receptors (Ry-R), transient receptor potential canonical ion channels [voltage-dependent Ca2^+^ channel (VDCC)]; PSD scaffolding proteins, such as Shank component of the NMDA receptor-associated PSD-95 complex. The binding to these molecules allows Homers to serve as a postsynaptic scaffold that crosslinks and, thus, regulates the functionality of the ligands. Long Homer isoforms additionally encode a C-terminal coiled-coil domain, which allows their multimerization at a specific site or subcellular compartment, such as the actin cytoskeleton. Synaptic multimerization of long Homer proteins probably regulates or facilitates signal transduction or cross-talk between target proteins. The short Homer isoform (Homer1a) lacks the coiled-coil domain and, therefore, cannot form multimers and functionally compete with the long Homer isoforms in binding postsynaptic signaling proteins. In this way, Homer1a acts as a natural dominant-negative regulator of the long Homers, causing rapid disruption of long Homer clusters and, consequently, affecting synaptic architecture.

The Homer family, a dendritically enriched scaffold protein group, is predominantly localized at the PSD in mammalian neurons where they contribute to the maintenance of synaptic functional integrity (52–54). The Homer genes can give rise to both constitutively expressed- and immediate early gene-products, which are induced by both physiological and abnormal neuronal activity (52). Alternative splicing results in multiple transcript variants, classified primarily into: long forms (Homer1b/c, Homer2a/b, and Homer3a/b), which represent the constitutively expressed isoforms; short forms, which represent the activity-dependent splice variants of the Homer1 gene (Homer1a and Ania3) (54, 55). All Homer proteins, through their N-terminal domain, bind a specific short proline-rich amino acid sequence found in several signaling proteins: Group1 of metabotropic glutamate receptors (mGluRs); Ca^2+^-signaling-related proteins, including phospholipase C-ß (PLCß), inositol 1,4,5-trisphosphate receptors, ryanodine receptors, transient receptor potential canonical ion channels; PSD scaffolding proteins, such as Shank component of the NMDA receptor-associated PSD-95 complex (52–54, 56–61). The binding to these molecules allows Homers to serve as a postsynaptic scaffold that crosslinks and, thus, regulates the functionality of the ligands (Figure 1) (62). Long Homer isoforms additionally encode a C-terminal coiled-coil (C-C) domain, which allows their multimerization at a specific site or subcellular compartment, such as the actin cytoskeleton (55). Synaptic multimerization of long Homer proteins probably regulates or facilitates signal transduction or cross-talk between target proteins; on the other hand, declustering of Homer is induced by an increase in intracellular Ca^2+^ concentration through NMDA receptors or voltage-dependent Ca^2+^ channels. In response to synaptic activity, reversible clustering and declustering of the Homer complex is probably an important mechanism used to alter the molecular composition within the complex, so that cross-talk signaling among cross-linked target proteins can be regulated (54). The short Homer isoforms lack the C-C domain and, therefore, cannot form multimers and functionally compete with the long Homer isoforms in binding postsynaptic signaling proteins (63). In this way, they act as a natural dominant-negative regulator of the long Homers, causing rapid disruption of long Homer clusters and, consequently, affecting synaptic architecture (53, 59, 64).

Due to their structural and functional presence in glutamate synapses, both immediate early gene and constitutively expressed Homer isoforms have been extensively involved in calcium signaling and behavioral pathologies associated with neuropsychiatric and neurodegenerative disorders, such as drug addiction, depression, anxiety, epilepsy and schizophrenia, Parkinson’s and Alzheimer’s disease (65–72). Some examples concern the deletion of Homer1, but not of Homer2, in the enhanced expression of certain anxiety- and depression-related behaviors in mice (73, 74); the blunted induction of Homer1a upon excessive synaptic activity as a putative mechanism in the etiology of epilepsy (75); Homer1 polymorphisms have a role in the etiology of schizophrenia, and Homer1-knockout (KO) mice exhibit behavioral and neurochemical abnormalities consistent with a schizophrenia-like phenotype; furthermore, the putative increase in Homer1a in the forebrain is indicated as part of the therapeutic and side-effect profiles of antipsychotic drugs (76–79). Homer proteins exhibit diverse regulatory roles in behavior and, among them, they seem to take part in sensitization to psychostimulant drugs, induction, and maintenance of excessive alcohol consumption, and addiction (80). In particular, low levels of Homer1 and Homer2 seem to represent an important molecular adaptation, associated with behavioral and neurochemical sensitivity to cocaine (81, 82). In this regard, a more comprehensive overview of Homer proteins implication in a wide variety of neuropsychiatric abnormalities can be found in Ref. (83). Recently, “novel” Homer2-interacting proteins have been detected, as part of the signaling cascade that is activated by the glutamatergic transmission, thus expanding the network of proteins that potentially contribute to the behavioral abnormalities associated with alcohol abuse (84, 85).

## Homer2 and Alcohol: The Interaction

Among the Homer isoforms, Homer2 has been shown to be critically important for plasticity in multiple models of alcohol abuse (44, 47–51, 86, 87). Homer2 expression is sensitive to alcohol experience. Even a first alcohol drink is sufficient to induce synaptic plasticity in NACsh D1-positive neurons where it increases synaptic expression of the scaffolding protein Homer2 and GluA1 5-methyl-4-isoxazolepropionate receptor subunit, *via* enhanced mammalian target of rapamycin complex 1-dependent RNA-to-protein translation (88). The increase in Homer2 levels has also been shown employing animal models of alcohol dependence and withdrawal, within brain regions relevant to addiction, and has been accompanied by functional alterations of glutamatergic synapses. In particular, a robust increase in Homer2 levels at 2 days, 2 weeks, and 2 months has been reported following withdrawal from chronic alcohol consumption within NAC, and was accompanied by a less enduring elevation in total mGluR1 and GluNR2b levels (48). Along with these results, short- and long-term withdrawal from intermittent and continuous alcohol consumption substantially increase Homer2a/b, mGluRs, as well as GluNR2a and GluNR2b subunits in the core (NACc), whereas slightly change GluNR2a and GluNR2b expression within the NACsh (89). Moreover, increased Homer2a/b, mGluR1, and GluNR2b expression have been detected in the central Amy (ceAmy), in both withdrawal groups, but not in the basolateral region, suggesting that withdrawal from chronic alcohol consumption produces enduring increase in the efficiency of postsynaptic glutamate receptor signaling that persists well beyond the time when signs of physical withdrawal have dissipated (89). Short-term withdrawal (24 h) from a chronic 30-day binge drinking period also increases Homer2a/b and PLCß expression within the ceAmy, concomitant with increased mGluR1 and GluN2B, not affecting the expression of correlated protein expression within the adjacent basolateral Amy (87). On the other hand, one recent research shows that, at 24 h withdrawal after 14 consecutive days of binge-drinking under 4-bottle drinking-in-the-dark (DID) procedures, both adult mice with prior history of binge-drinking during adolescence and adult mice without drinking history display a significant decrease in ceAmy Homer2b expression (90). At the present time, it remains to clarify whether or not the opposite trend of findings reflects procedural differences related to the total duration of alcohol-access or to the number of bottles presented during alcohol-access. Finally, evidence exists on how binge alcohol drinking under the scheduled high alcohol consumption (SHAC) (86) and the DID procedures (49) affect the expression of glutamate signaling-related proteins within brain regions implicated in the neurobiology of alcoholism. SHAC procedure doubles NAC Homer2a/b expression, along with a smaller, albeit significant, increase in NAC levels of GluNR2a and GluNR2b (86); limited access to alcohol drinking under the DID procedure upregulates NACsh Homer2a/b levels, concomitant with increases in mGluR5 and GluNR2b (49). It is possible to theorize that an alcohol-induced increase in Homer2 signaling and, thus, in glutamate receptor plasticity, within both the “motor” (NACc) and “motive” (NACsh) striatal regions, and in ceAmy, is a pharmacodynamic response to alcohol that likely contributes to the propensity to further consume excessive amounts of alcohol (44, 84) (Table 1). In this regard, converging data derived from both human (51) and animal (44, 47–50) studies implicate Homer gene products in behavioral sensitivity to alcohol and addiction vulnerability. Researchers adopted numerous strategies to examine the critical role for Homer2 in maintaining alcohol consumption and promoting addiction. Virus-mediated NAC Homer2 overexpression augmented various aspects of alcohol-related behavior in rodents (48, 50). In more detail, Szumlinski et al. (48) have shown that viral transfection of NAC neurons with an adeno-associated virus (AAV) carrying Homer2b in alcohol-preferring C57BL6/J (B6) inbred mice enhances behavioral output for alcohol in an operant self-administration paradigm and facilitates the expression of an alcohol-conditioned place-preference. Moreover, following an acute alcohol injection, NAC Homer2 overexpression facilitated NAC glutamate- and DA release and augmented alcohol-induced DA and glutamate sensitization. Since ceAmy dysfunction is critically implicated in alcohol withdrawal-induced negative affect, changes in Homer2 protein levels within this region closely align with the manifestation of a negative affective state, including the incubation of an adverse condition in those with a history of adolescent-onset binge-drinking. AAV Homer2-cDNA infusion reversed the hyper-anxious phenotype in adolescent-onset alcohol-drinking mice during protracted withdrawal and reduced subsequent alcohol consumption. Conversely, Homer2-cDNA was anxiogenic and significantly increased drinking in alcohol-inexperienced mice (91). Thus, Homer2 overexpression is functionally correlated to an increase in alcohol potency and efficacy to elicit reward, through an enhancement in both appetitive and consummatory alcohol-related behavior, mediated by the role of Homer2 expression in gating the negative reinforcing properties of alcohol that drive excessive intake. Conversely, Homer2 deletion [knockout (KO)], knockdown, or blockade result in an alcohol-avoiding phenotype (47, 49, 50). In particular, Homer2 knockout (H2KO) mice display a lower preference for alcohol versus water in the preference test, than the wild-type (WT) mice, and exhibit place aversion after alcohol-place conditioning. It appears, therefore, that H2KO mice display a deficient behavioral plasticity to alcohol. This is accompanied by inhibition of the raise in extracellular DA and glutamate release within the NAC, after repeated injections of alcohol (47). The Homer2 scaffolding is essential to observe the modulatory effects of mGlu1/5-protein kinase C (PKC)ε and PLC signaling on voluntary alcohol intake. As consistently described, whereas the infusion of mGluR1 antagonist into the NACsh, the intra-ceAmy infusion of mGluR1, mGluR5, and PLC inhibitors (87) and inhibition of PKCε translocation within both ceAmy and NAC (92) dose-dependently reduce alcohol consumption in B6 and Homer2 WT mice, this effect is not present in H2KO animals (44, 87). Even knocking-down Homer2 levels within the NACsh (49) and ceAmy (87) affect alcohol consumption: it has been shown that Homer2b knockdown significantly reduced alcohol intake in the DID model and, later on, under the DID four-bottle-choice procedure at 40% alcohol.

**Table 1:** The effects of alcohol treatments in rodents on Homer2 isoform levels.

Procedure	Animal	Tissue	Detection technique	Effect on Homer2 levels	Reference
Protracted alcohol withdrawal after chronic alcohol consumption	C57BL/6J (B6) mice	NAC	Immunoblotting	Robust increase	Szumlinski et al. (48)

Short- versus long-term withdrawal (SW or LW) from ethanol consumption	P rats	Nucleus accumbens shell (NACsh)	Immunoblotting	Limited changes	Obara et al. (89)
		ceAmy and NACc	Immunoblotting	Substantial changes	

Scheduled high alcohol consumption (SHAC) procedure	C57BL/6J (B6) mice	NAC	Immunoblotting	Doubling	Cozzoli et al. (86)

Drinking-in-the-Dark (DID) procedure	C57BL/6J (B6) mice	NACsh	Immunoblotting	Upregulation	Cozzoli et al. (49)

Short-term withdrawal from a chronic history of binge drinking	C57BL/6J (B6) mice	ceAmy	Immunoblotting	Increase	Cozzoli et al. (87)

4-bottle DID procedure	C57BL/6J (B6) mice	ceAmy	Immunoblotting	Decrease	Lee et al. (90)

Finally, complementing prior findings from mouse models of alcohol vulnerability, data from Haider and colleagues indicate that Homer2 within the NACc bidirectionally regulates alcohol intake in both alcohol-preferring (P) and Wistar rats. They have employed an AAV strategy to overexpress and knock down Homer2b within the rat NAC, under continuous-access alcohol drinking. Homer2b overexpression elevates, while Homer2b knockdown reduces alcohol intake in both lines in a dose-dependent manner (50). Finally, the first study to examine the association between human alcohol use and the glutamatergic pathway-related genes shows that, among the others, genetic variations of Homer2 are associated with increased alcohol consumption in the Detroit Neighborhood Health Study (51).

These relevant findings point to Homer2-mediated organization of the glutamate synapse as a regulator of acute alcohol sensitivity and vulnerability (Table 2). These alterations, accompanied by spine structural abnormalities, are mainly restricted to the withdrawal phase of alcohol dependence, suggesting their relevance in the genesis of psychological and emotional signs and/or symptoms of acute abstinence (3).

**Table 2:** The effect of the alterations of Homer2 isoform on the alcoholic phenotype.

Treatment	Test	Animal	Tissue	Data	Reference
Adeno-associated virus (AAV) carrying Homer2b	Operant paradigm	C57BL/6J (B6) mice	NAC	Enhanced behavioral output for alcohol	Szumlinski et al. (48)
	Conditioned place-preference			Up-expression of an alcohol-conditioned place-preference	
				Increase in glutamate and dopamine (DA) release	
	Ethanol place-conditioning test	Homer2 knockout (H2KO) mice	NAC	Strong preference for water	Szumlinski et al. (47)
	Ethanol versus water preference test			Place aversion	
				Locomotor depression; no behavioral adaptation	
				No increase in extracellular levels of DA and glutamate	
	Drinking-in-the-Dark (DID) procedure	H2KO mice	Nucleus accumbens shell (NACsh)	Significant reduction in alcohol intake	Cozzoli et al. (49)

Infusion of mGluR1 antagonist	DID procedure	H2KO mice	NACsh	Reduction of alcohol consumption	Lum et al. (44)
		C57BL/6J mice		No reduction in alcohol consumption	
AAV-shRNA-Homer2b	4-bottle DID procedure	Homer knockdown	ceAmy	Significant reduction at the highest alcohol concentrations	Cozzoli et al. (87)
		AAV-GFP-infused controls		No reduction in alcohol consumption	
Infusion of mGluR1, mGluR5 and PLC inhibitors	DID procedure	H2KO mice	ceAmy	No effect in alcohol intake	Cozzoli et al. (87)

AAV-cDNA-Homer2b	Continuous alcohol access procedure	Alcohol-preferring P rats and Wistar rats	NACc	Elevation in alcohol intake	Haider et al. (50)

AAV-shRNA-Homer2b	Continuous alcohol access procedures	Alcohol-preferring P rats and Wistar rats	NACc	Reduction in alcohol intake	Haider et al. (50)

Inhibition of protein kinase Cε translocation	DID and SHAC procedures	H2KO mice	NAC and ceAmy	No effect in alcohol intake	Cozzoli et al. (92)
		Homer2 wild-type mice		Inhibition of alcohol intake	

AAV-cDNA-Homer2	3-bottle DID procedure	Adolescent-onset alcohol-drinking mice	ceAmy	Reduction in alcohol consumption	Lee et al. (91)
		Alcohol-inexperienced mice		Significant increase in alcohol consumption	

## Conclusion

Data reported provide evidence that alcohol drinking produces an enduring upregulation of the expression of specific components of the glutamate signaling cascade, in particular elevations in Homer2 levels. This neuroadaptation is proposed to contribute to the hyper-glutamatergic signaling that mediates alcohol-induced neuroplasticity within the NAC and Amy and promotes alcohol-related behaviors, sustaining the relapsing nature of alcohol abuse. Thus, the interaction between Homer2 and alcohol can be defined as a mutual relation: alcohol consumption enhances Homer2 protein isoforms expression within NAC and the extended Amy, cerebral areas where, in turn, Homer2 is able to mediate alcohol rewarding properties, leading to further alcohol consumption. This interaction is intertwined within the addiction cycle of alcohol: from the initial, voluntary, and single use, to the further repeated consumption and abuse, to addiction and withdrawal when increase in Homer2 signaling strength may promote relapse and further abuse.

The integrity of the PSD is important for normal spine morphology and activity-dependent adaptations of synaptic strength (93). A central question in neuroscience is how dendritic spines and synapses can be structurally and functionally modified to support experience-dependent changes in neuronal connectivity (64), leading to alcohol dependence. In this regard, the perturbation in the homeostasis of scaffolding proteins, first and foremost Homer2, has been associated with abnormal neuronal orientation, morphology, and axonal connections, which need to be further investigated in alcohol use disorder, in the early and late life stages. In particular, the assessment of the role of Homer proteins in vulnerability to drug abuse constitutes an unexplored research venue. Perinatal alcohol exposure (94) could alter Homer2 protein expression pattern, when the developing central nervous system is extremely sensitive to pharmacological and environmental manipulations (95–97).

Overall, these findings bridge preclinical and clinical knowledge regarding a role for Homer2 isoforms in regulating addiction vulnerability. Such evidence may help the detection of strategic molecular components that prompt alcohol-induced functional and behavioral disarrangement as targets for future innovative treatment options.

## Author Contributions

VC, AB, GL, AC, and CC wrote major parts of the article. All authors critically reviewed and edited the article. The mini review article was written based on the expertise of the authors, who have sourced the literature on PubMed and Google Scholar.

## Conflict of Interest Statement

The authors declare that the research was conducted in the absence of any commercial or financial relationships that could be construed as a potential conflict of interest.

## Abbreviations

AMPAR, 5-methyl-4-isoxazolepropionate receptor; SER, smooth endoplasmic reticulum; GKAP, guanylate kinase-associated protein; GRIP, glutamate receptor interacting protein; mGluR, metabotropic glutamate receptor; NMDAR, *N*-methyl-d-aspartate receptor; VDCC, voltage-dependent Ca^2+^ channel.
